# Duplex EIS Sensor for *Salmonella Typhi* and *Aflatoxin B1* Detection in Soil Runoff

**DOI:** 10.3390/bios15100654

**Published:** 2025-10-01

**Authors:** Kundan Kumar Mishra, Krupa M Thakkar, Sumana Karmakar, Vikram Narayanan Dhamu, Sriram Muthukumar, Shalini Prasad

**Affiliations:** 1 Department of Bioengineering, University of Texas at Dallas, Richardson, TX 75080, USA; kundan.mishra@utdallas.edu (K.K.M.); krupa.thakkar@utdallas.edu (K.M.T.); sumana.karmakar@utdallas.edu (S.K.); 2 EnLiSense LLC, 1813 Audubon Pondway, Allen, TX 75013, USA; vikram@enlisense.com (V.N.D.); sriramm@enlisense.com (S.M.)

**Keywords:** electrochemical impedance spectroscopy, *Salmonella*, *Aflatoxin*, immunosensor

## Abstract

Monitoring contamination in soil and food systems remains vital for ensuring environmental and public health, particularly in agriculture-intensive regions. Existing laboratory-based techniques are often time-consuming, equipment-dependent, and impractical for rapid on-site screening. In this study, we present a portable, non-faradaic electrochemical impedance-based sensing platform capable of simultaneously detecting *Salmonella Typhimurium* (*S. Typhi*) and *Aflatoxin B1* in spiked soil run-off samples. The system employs ZnO-coated electrodes functionalized with crosslinker for covalent antibody immobilization, facilitating selective, label-free detection using just 5 µL of sample. The platform achieves a detection limit of 1 CFU/mL for *S. Typhi* over a linear range of 10–10^5^ CFU/mL and 0.001 ng/mL for *Aflatoxin B1* across a dynamic range of 0.01–40.96 ng/mL. Impedance measurements captured with a handheld potentiostat were strongly correlated with benchtop results (R^2^ > 0.95), validating its reliability in field settings. The duplex sensor demonstrates high precision with recovery rates above 80% and coefficient of variation below 15% in spiked samples. Furthermore, machine learning classification of safe versus contaminated samples yielded an ROC-AUC > 0.8, enhancing its decision-making capability. This duplex sensing platform offers a robust, user-friendly solution for real-time environmental and food safety surveillance.

## 1. Introduction

There are numerous contaminants that can infiltrate agricultural produce and water sources via soil run-off, posing a significant threat to the well-being of humans and animals. In particular, *Salmonella Typhimurium* (*S. Typhi*) and *Aflatoxin B1* (AFB_1_) are amongst the most perilous contaminants [1,2,3,4,5]. AFB_1_ is an extremely toxic mycotoxin produced by *Aspergillus* fungi that is commonly found in crops and contaminated soil. Soil-bound AFB_1_ can aerosolize or leach into runoff, leading to the pollution of crops and water sources. Ingestion of AFB_1_ can have detrimental effects on multiple organ systems such as the digestive, circulatory, respiratory, and reproductive systems. Specifically, lethal amounts of AFB_1_ can result in liver cancer [6,7]. *S. Typhi* is a Gram-negative, rod-shaped bacterium that is transmitted via fecal-contaminated water. Additionally, it has been linked to surface water and agricultural irrigation systems. Exposure of *S. Typhi* can lead to typhoid fever, a life-threatening infection that can result in prolonged fever, sore throat, cough, abdominal pain, rash, diarrhea, and enlargement of the spleen and liver [8,9,10,11].

Importantly, AFB_1_ and *S*. *Typhi* can co-occur in the same environmental sample, particularly in soil run-off and irrigation water. Soil colonized by Aspergillus fungi contributes mycotoxins such as AFB_1_, while agricultural and urban run-off contaminated with human or animal waste introduces *S. Typhi*. During flooding or heavy rainfall, both contaminants may be mobilized together into crops, irrigation systems, or water reservoirs, creating compounded risks to food safety and public health. Thus, simultaneous monitoring of these hazards is highly relevant in real-world scenarios where microbial and chemical contaminants coexist in complex environmental matrices. Despite the regulatory limits imposed by agencies such as the U.S. Food and Drug Administration (FDA) and the European Union (EU), the main challenge remains the lack of effective field-deployable tools for concurrent detection of microbial and chemical hazards. For AFB_1_, the FDA and EU permit up to 20 ppb and 2 ppb, respectively, for human consumption, while both agencies strictly prohibit the presence of *S. Typhi* in food and water samples [2,12].

Traditional detection methods for *S. Typhi* and AFB_1_ rely on conventional laboratory assays: chromatographic methods (e.g., HPLC-MS) or immunoassays (e.g., ELISA) for AFB_1_, and culture or molecular (PCR) methods for *Salmonella* [13,14,15]. Although acknowledged as highly sensitive, these techniques are time-consuming, labor-intensive, demand expensive equipment, and require trained laboratory technicians. For instance, chromatography with QuEChERS sample preparation is a common method for soil residue analysis. The addition of chromatography steps (such as column preparation and sample extraction) can make the detection process quite laborious and tedious. Culture and/or PCR for typhoid detection requires long periods of time and a pre-enrichment protocol. This hinder rapid field decisions, deeming it unrealistic for on-site usage. On the other hand, electrochemical impedance spectroscopy (EIS) offers label-free, rapid biosensing with minimal sample preparation [16,17,18]. EIS detects biomolecular binding at electrode interfaces by quantifying changes in charge-transfer resistance or capacitance. Integration of biological recognition, such as antibodies or aptamers, on nanostructured electrodes yields high selectivity and sensitivity. Notably, ZnO nanostructures provide a large surface area, superior electron mobility, and biocompatibility. These properties help ameliorate antibody loading and electron transfer, therefore lowering detection limits and boosting EIS signaling. Supplementary Table S2 presents a comparative overview of various immunosensors, emphasizing that many existing label-free approaches lack direct sensitivity toward *S. Typhi* and AFB_1_.

To address the drawbacks of conventional analytical techniques—such as their high cost, complex procedures, bulky instrumentation, and reliance on trained personnel, there is an increasing demand for rapid, sensitive, and field-deployable detection systems, particularly for complex matrices like soil run-off. In response to this need, we present a duplex electrochemical sensing platform leveraging Electrochemical Impedance Spectroscopy (EIS) for the simultaneous detection of *S. Typhi* and AFB_1_. This platform employs antibody-functionalized electrodes integrated using a bifunctional crosslinker, ensuring both high specificity and stable immobilization of target analytes. Operating in non-Faradaic mode, EIS allows label-free, real-time detection by monitoring interfacial capacitance changes, eliminating the requirement for redox agents or labeling steps. The sensor achieves high sensitivity and low detection limits for both *S. Typhi* and AFB_1_, demonstrating effective performance even in environmentally challenging matrices such as soil run-off. The system is optimized to operate at 200 Hz, where impedance changes are most responsive to biomolecular interactions at the sensor interface. This frequency optimization enhances the sensor’s ability to selectively identify AFB_1_ among structurally similar mycotoxins while also detecting pathogenic bacteria like *S. Typhi* concurrently, with minimal cross-reactivity. The novelty of this work lies in the true duplex configuration, where two independent sensing elements are integrated on a single platform to enable simultaneous detection of a bacterial pathogen and a toxin in real time. By combining frequency-tuned, non-Faradaic impedance measurements with antibody-functionalized surfaces, the system achieves both high sensitivity and selectivity in complex matrices. This advancement goes beyond existing single-analyte EIS approaches, offering a scalable and field-deployable solution for food and environmental safety monitoring. Furthermore, the robust and consistent performance in soil-based matrices highlights the platform’s suitability for environmental applications beyond conventional food analysis. The core innovation of this study lies in its duplex, non-Faradaic impedance approach, which enables simultaneous detection and target discrimination through distinct circuit responses. This makes the system a portable, cost-effective, and user-friendly alternative to traditional laboratory-based methods—offering a significant advancement toward real-time, on-site monitoring of both microbial and chemical hazards in support of environmental safety and public health initiatives.

## 2. Materials and Methods

### 2.1. Materials and Reagents

The *Aflatoxin B1* and *Salmonella Typhi* antibodies were purchased from Invitrogen (Carlsbad, CA, USA), and the crosslinker DTSSP (3,3′dithiobis(sulfosuccinimidyl propionate)) and Phosphate-buffered saline (PBS) were obtained from Thermo Fisher Scientific Inc. (Waltham, MA, USA). All chemicals were of analytical grade and used without further purification. Deionized water (resistivity ≥18 MΩ·cm) was obtained from a Milli-Q water purification system (MilliporeSigma, Burlington, MA, USA). These materials are necessary for the sensor modification process to enable accurate detection of analytes within a complex matrix, such as soil runoff. All chemicals involved in the experiment process are laboratory-grade and ultra-purified to avoid additional purification procedures and conserve time and resources.

### 2.2. Electrode Surface Modification and Testing Process

To begin the sensor modification process, the surface of the electrode was rinsed with PBS to remove any debris or impurities that could hinder the detection mechanism. Next, a cocktail was created containing 6 mM of the DTSSP crosslinker with 10 μg/mL *S. Typhi* or AFB_1_ antibodies. Then, the DTSSP crosslinker and antibody mixture is placed at room temperature for 30 min in a dark environment to affirm that light will not interfere with the binding of the antibodies to the crosslinker. Subsequently, this solution is then evenly spread across the electrode surface and incubated for 30 min at room temperature. The DTSSP crosslinker facilitates a covalent interaction between the antibodies and the electrode surface. The DTSSP crosslinker binds to the coating on the electrode surface. The NHS ester group present in the DTSSP crosslinker reacts with the amine groups on the antibodies. This attachment helps stabilize the antibody on the electrode surface. After the 30-min incubation period, the solution was removed, and the electrode surface was rinsed with PBS to remove any unbound reagents from the electrodes. Following this, the electrode surface was treated with T20 (PBS) blocking buffer, referred to here as “superblock,” and incubated at room temperature for 10 min in the dark to prevent non-specific binding. The blocking buffer was then aspirated, and the electrode surface was rinsed again with PBS. Subsequently, the chips were put in a lyophilization machine to freeze to −18 °C, following vacuum pressurization at 5 pascals for 25 min. This process helps conserve the durability and reliability of the sensor, guaranteeing proper detection of harmful contaminants in environmentally diverse samples. To begin the testing process, 5 μL of the *S. Typhi* or AFB_1_ sample was placed on the modified electrode surface. The sensor was placed in a 5-min incubation period to enhance antibody–antigen interaction. When the incubation period was over, electrochemical impedance spectroscopy (EIS) was used to analyze the analyte detection process taking place between the antigen in the sample and the antibodies adhered on the electrode surface by the DTSSP crosslinker. The impedance values were conducted within a frequency range from 1000 Hz to 80 Hz with a 10 mV AC bias.

### 2.3. Sensor Design

The biosensor is built on a three-electrode configuration, consisting of a gold working electrode (WE), a gold reference electrode (RE), and a carbon counter electrode (CE). To improve the surface-to-volume ratio of the working electrode and enhance biomolecule immobilization, a thin semiconducting layer was deposited. This semiconducting layer was applied using sputter deposition, a physical vapor deposition method well known for producing uniform, adherent, and stable coatings on metallic substrates. Electrochemical impedance spectroscopy (EIS) was employed as the primary analytical technique to monitor antigen–antibody interactions on the modified electrode surface. The sensor array includes 2 independent WEs, REs, and CEs, enabling simultaneous and multiplexed impedance measurements.

### 2.4. Sample Preparation and Duplex Sensor Design

**Figure 1:**
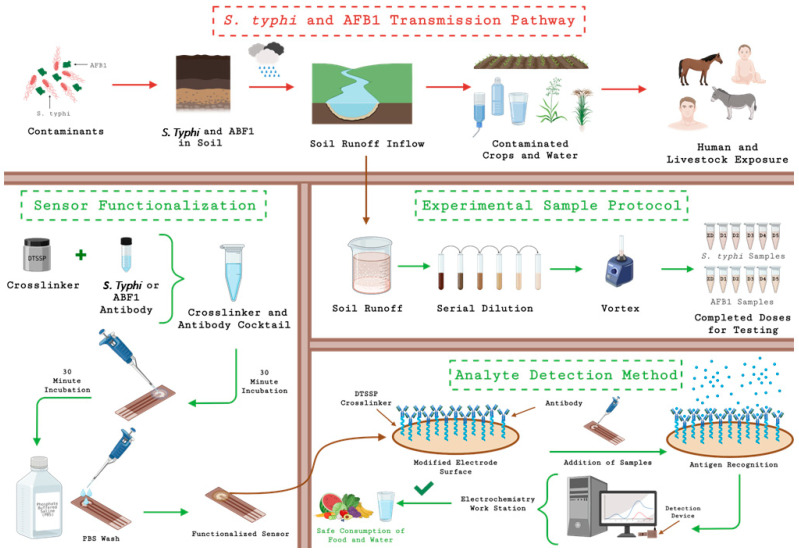
Schematic representation of the sample matrix preparation and electrochemical sensing workflow.

Soil runoff samples were simulated by mixing topsoil with PBS and vortexing, then centrifuging to remove particulates. Known concentrations of *S. Typhi* and AFB_1_ were spiked into the supernatant to evaluate sensor response. Figure 1 schematically illustrates the workflow. The duplex sensor platform consists of a Au substrate is coated with ZnO nanostructures (via sputtering or nanowire growth) to increase the surface area. The ZnO-coated electrodes are first treated with DTSSP, a thiol-containing cross-linker that forms a self-assembled monolayer and provides NHS-ester groups. Each channel is then separately incubated with one type of antibody (anti-AFB_1_ or anti-*Salmonella*), which covalently bind via amide linkage to DTSSP. Finally, a blocking step prevents nonspecific adsorption. This immuno-modified sensor can then capture analyte from the sample when exposed. The use of ZnO is key: its high intrinsic electron mobility and biocompatibility facilitate fast charge transfer and robust antibody immobilization. As illustrated, antigen binding alters the interfacial impedance (primarily the charge-transfer resistance) on each channel. Thus, EIS measurements can quantify each analyte separately in one assay. This detection design offers major advantages in environmental monitoring: it combines toxin and pathogen testing in one device, reducing sample processing and allowing a comprehensive safety assessment of runoff.

## 3. Results and Discussion

### 3.1. Electrochemical Impedance Spectroscopy (EIS) Analysis and Spike and Recovery Assessment

**Figure 2:**
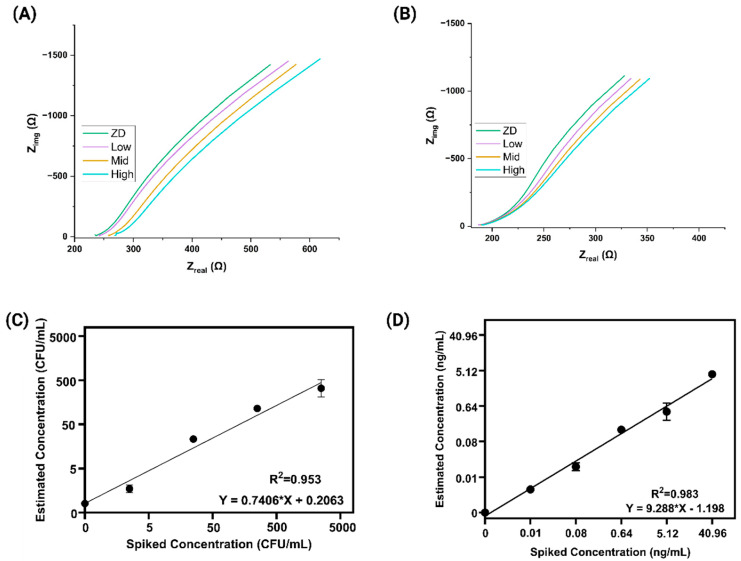
Nyquist plots for *Salmonella Typhi* (**A**) and *Aflatoxin B1* (**B**) in the duplex sensor platform, respectively, at ZD (control), low, mid and high spiked concentrations in soil runoff. (**C**,**D**) Represent the correlation between spiked and estimated concentrations on the 2-plex sensor, with spiking ranges of 10–10^5^ CFU/mL for *Salmonella Typhi* (**C**) and 0.01–40.96 ng/mL for *Aflatoxin B_1_* (**D**) in soil runoff, respectively.

To assess the electrochemical performance of our tailored biosensor, we employed Electrochemical Impedance Spectroscopy (EIS) for the recognition of *S. Typhi* and AFB_1_ in soil run-off samples. Through this modification, electrochemical measurements were gathered via EIS with a 10 mV AC bias [19,20,21,22]. Binding of the pathogen and toxin [23,24] analytes to their respective probes on the sensor surface caused measurable changes in the electrical double layer. The sensor surface was functionalized using 3,3′-dithiobis(sulfosuccinimidyl propionate) (DTSSP) to ensure effective antibody immobilization. Successful antibody conjugation was confirmed via FTIR in our previous work [25,26]. In addition, cyclic voltammetry (CV) was performed to characterize the electrode surface at each modification step, showing distinct current responses for bare, DTSSP-modified, and antibody-functionalized electrodes, thereby validating successful surface modification (Supplementary Figure S1). The corresponding calibrated dose–response (CDR) plots for varying analyte concentrations in potable water are presented in Supplementary Figure S2. We evaluated the sensor’s response using electrochemical impedance spectroscopy (EIS) to assess the binding interaction of the analytes with the functionalized electrode surface. Figure 2A presents Nyquist plots (imaginary impedance Z″ vs. real impedance Z′) for *S. Typhi* at concentrations of 10, 10^3^, and 10^5^ CFU/mL in spiked soil runoff. A clear shift in the impedance trace is observed as the concentration increases, with the plot moving progressively toward the real axis [27,28,29,30,31]. This trend reflects changes in the electrical double layer at the electrode interface due to increased bacterial binding, which alters interfacial capacitance and impedes ion mobility near the surface. Figure 2B shows a similar dose-dependent trend for AFB_1_, tested at concentrations of zero dose (ZD), low (0.01 ng/mL), mid (0.64 ng/mL), and high (40.96 ng/mL). As AFB_1_ concentration increases, the Nyquist trace shifts closer to the real axis, indicating modulation of the electrical double layer [28,32,33,34,35,36]. This shift results from enhanced analyte–antibody interactions at the electrode surface, which alter interfacial capacitance and restrict ion movement near the interface. In both figures, the systematic rightward shift of the Nyquist lines confirms that the sensor effectively distinguishes increasing analyte concentrations through impedance changes. These findings highlight the sensor’s sensitivity and suitability for detecting both microbial and chemical contaminants in complex environmental samples [37,38].

Spike and recovery studies were conducted to evaluate the sensor’s accuracy, precision, and applicability in detecting *S. Typhi* and AFB_1_ in complex environmental matrices like soil runoff. In this experiment, known concentrations of the two analytes were spiked into the matrix to simulate real-world contamination, and the sensor’s response was quantified using the calibrated dose–response (CDR) approach.

The recovery percentage was calculated using the standard formula:

Percentage Recovery (%) = (Detected Concentration/Spiked Concentration) × 100

Figure 2C shows the spike-and-recovery analysis for *S. Typhi*, where different concentrations (10, 10^2^, 10^3^,10^4^, and 10^5^ CFU/mL) were spiked into the matrix. The detected values were plotted against the known spiked concentrations, and a strong correlation was observed with an R^2^ value of 0.953, confirming good linearity and consistency in detection. Similarly, Figure 2D presents the corresponding spike-and-recovery plot for AFB_1_, with concentrations of 0.01, 0.08, 0.64, 5.12, and 40.96 ng/mL. The estimated concentrations closely matched the spiked levels, yielding a high correlation coefficient (R^2^ = 0.9837), indicating excellent agreement between actual and measured values. Each test was performed with N = 8 replicates to ensure statistical significance and repeatability. These results validate the sensor’s reliability and robustness, demonstrating its effectiveness in accurately detecting both bacterial and toxin contaminants in complex matrices like soil runoff.

### 3.2. Correlation Study Between the Benchtop and Portable Device

Following the successful development of the electrochemical sensor for detecting *S. Typhi* and AFB_1_ in soil runoff, we conducted a correlation study to assess the performance of the portable device relative to a benchtop laboratory instrument. The primary objective was to validate the accuracy and reliability of the portable device for real-world, field-deployable applications. In this study, impedance data from both the portable device and the benchtop system were compared using controlled samples spiked with known concentrations of *S. Typhi* and AFB_1_.

**Figure 3:**
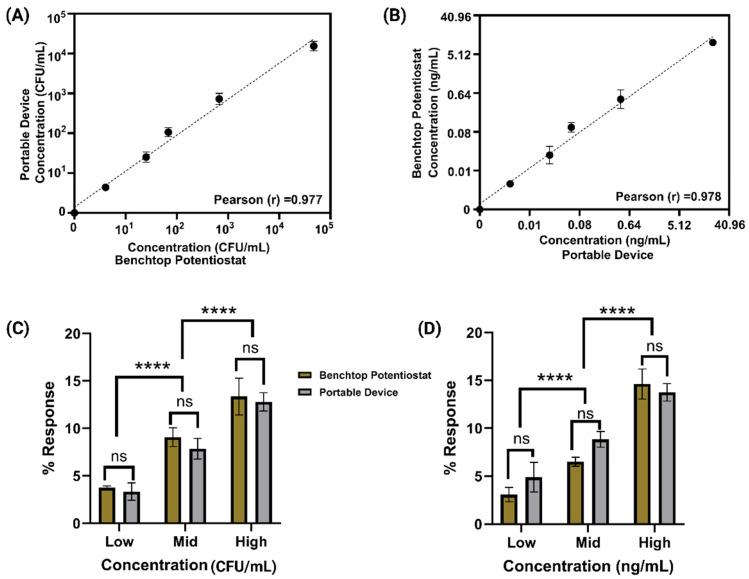
(**A**,**B**) Correlation analysis between the portable and benchtop devices across five concentrations for *S. Typhi* and AFB_1_, demonstrating a strong linear agreement. (**C**,**D**) Comparative dose analysis using paired *t*-tests for both devices (ns: no significant difference at corresponding concentrations; **** indicates significant differences between low, mid, and high concentration levels).

A Pearson correlation analysis was performed to evaluate the linearity and consistency between the two platforms. The portable device demonstrated strong agreement with the benchtop instrument, confirming its suitability for reliable field measurements. The correlation coefficients (R^2^) were 0.977 for *S. Typhi* and 0.978 *for* AFB_1_, as shown in Figure 3A,B. These high R^2^ values indicate that the portable device delivers reproducible and accurate results comparable to those from a standard laboratory setup.

Additionally, a paired *t*-test was conducted to assess the statistical significance of differences between the two systems at corresponding concentrations. No statistically significant differences were observed, with *p*-values of 0.71 for *S. Typhi* and 0.62 for AFB_1_, as shown in Figure 3C,D. These results support the reliability of the portable platform for on-site testing. However, significant differences were observed when comparing responses between low, mid, and high dose levels, with *p*-values of 0.0001 for both *S. Typhi* and AFB_1_, indicating concentration-dependent variability that is important for practical deployment and calibration strategies. Overall, this correlation study demonstrates that the portable device is capable of delivering accurate, reproducible, and field-relevant results for the simultaneous detection of microbial and chemical contaminants in complex environmental samples.

### 3.3. Cross Reactivity Repeatability and Reproducibility

**Figure 4:**
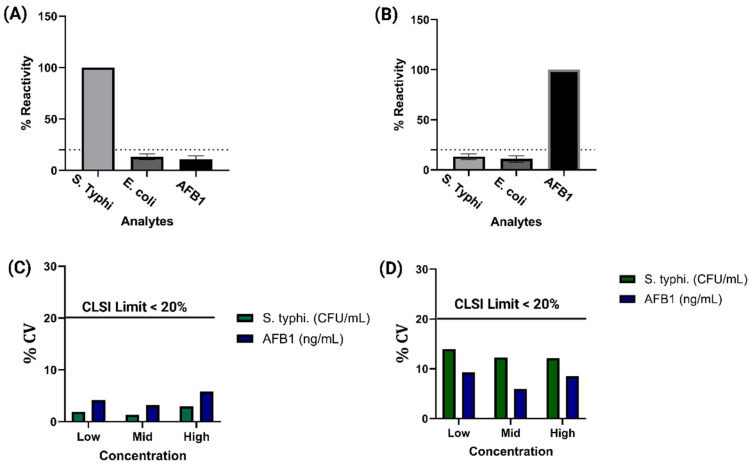
(**A**,**B**) Cross-reactivity analysis for *S*. *Typhi* and AFB_1_-modified sensors tested with mixed analyte cocktails, showing minimal non-specific response. (**C**,**D**) Repeatability and reproducibility assessment across multiple concentrations, with %CV values below 20% confirming consistent sensor performance.

For a Duplex electrochemical sensor developed for on-site food safety monitoring, ensuring high specificity and minimal cross-reactivity is essential to maintain reliable and accurate detection in complex sample matrices. In field applications, a wide range of interfering substances, including structurally similar contaminants, may coexist with target analytes, potentially affecting the sensor’s performance. To evaluate the specificity of the sensor, we conducted a systematic cross-reactivity study using soil run-off as the test matrix, spiked with combinations of structurally related analytes. Figure 4A illustrates the response of the sensor modified with *S. Typhi* antibodies in a cocktail containing *S. Typhi*, *E. coli* O157:H7 and AFB_1_. The impedance change remained under 10%, indicating minimal cross-reactivity. Similarly, [39,40,41] Figure 4B shows the response of the AFB_1_-antibody modified sensor in the same cocktail, again demonstrating less than 10% change in impedance [42,43]. To further assess selectivity against structurally similar mycotoxins, we evaluated the cross-reactivity of the AFB_1_ sensor in the presence of *Aflatoxin M1* (AFM_1_), as shown in Supplementary Figure S3. The response remained below 10%, confirming that the platform can effectively discriminate AFB_1_ even from closely related analogs. Collectively, these results demonstrate the high selectivity and robustness of the duplex sensor in distinguishing its specific targets, even in complex mixtures containing multiple microbial and chemical interferents.

To further assess the robustness and reliability of the sensor, repeatability and reproducibility tests were performed. Intra-assay (within-run) repeatability was measured by analyzing multiple replicates of each concentration in a single assay, while inter-assay (between-run) reproducibility was evaluated by repeating the measurements across different days and conditions. As depicted in Figure 4C, the coefficient of variation (%CV) for inter-assay results across all antibiotics and concentrations remained below 20%, meeting the acceptable threshold defined by the Clinical and Laboratory Standards Institute (CLSI) [44,45]. Likewise, Figure 4D shows that intra-assay variability also stayed under 20% for all tested conditions. Together, these findings highlight the sensor’s strong analytical performance, characterized by high specificity, minimal cross-reactivity, and consistent reproducibility—critical attributes for reliable toxin detection in field-deployable food safety monitoring platforms.

### 3.4. Classifier Model Study

**Figure 5:**
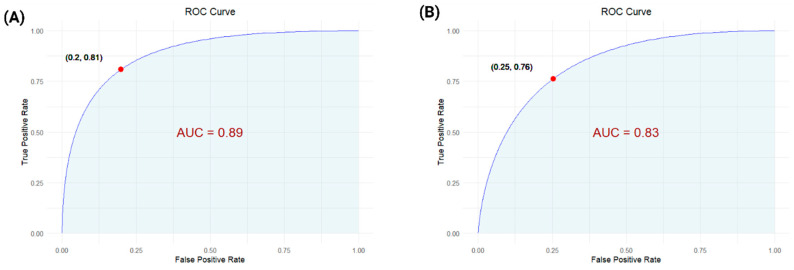
ROC curves for (**A**) *S. Typhi* and (**B**) AFB_1_ detection, showing high classification accuracy with AUCs of 0.89 and 0.83, respectively, validating the sensor’s diagnostic performance.

To assess the predictive performance of the electrochemical sensor platform, a Classifier Model Study was conducted using Receiver Operating Characteristic (ROC) curve analysis for both *S. Typhi* and AFB_1_. This study was designed to quantitatively evaluate the sensor’s ability to distinguish contaminated samples from non-contaminated controls, thereby linking experimental measurements to real-world diagnostic relevance. ROC analysis plots sensitivity versus 1-specificity, enabling identification of the optimal threshold for classification. For *S. Typhi* detection, the classifier achieved a sensitivity of 90.21%, specificity of 89.01%, Positive Predictive Value (PPV) of 81%, and Negative Predictive Value (NPV) of 91%, with an Area Under the Curve (AUC) of 0.89 (Figure 5A), indicating excellent predictive accuracy. For AFB1, the sensor demonstrated a sensitivity of 88.45%, specificity of 90.23%, PPV of 80.2%, and NPV of 88.96%, with an AUC of 0.83 (Figure 5B), confirming strong classification performance [46,47,48,49]. These results demonstrate the robustness of the classifier and highlight the sensor’s reliability for rapid, on-site detection, reinforcing the overall research objective of developing a duplex, field-deployable food safety monitoring platform.

## 4. Conclusions

We developed a portable, electrochemical impedance-based duplex sensor for the simultaneous detection of *S. Typhi* and AFB_1_ in soil run-off samples, achieving low detection limits with high specificity using just 5 µL of sample and providing results in under 5 min. The sensor integrates target-specific antibodies via a bifunctional crosslinker for stable immobilization and minimal cross-reactivity, ensuring consistent performance across complex environmental matrices. By optimizing the sensing response at 200 Hz, the system leverages frequency-resolved interfacial charge modulation to discriminate between *S. Typhi* and structurally similar bacterial strains, as well as between AFB_1_ and related mycotoxins. This frequency-targeted tuning enables label-free, real-time detection without the need for sample preprocessing or external reagents. With its rapid response, multi-analyte capability, and field-readiness, the platform holds strong promise for real-time environmental and food safety monitoring. Future work will focus on expanding analyte coverage, enhancing sensor robustness for long-term deployment, and validating system performance across diverse environmental conditions and matrices.

## Data Availability

The data that support the findings of this study are available on request from the corresponding author. The data are not publicly available due to privacy or ethical restrictions.

## Supplementary Materials

The following supporting information can be downloaded at: https://www.mdpi.com/article/10.3390/bios15100654/s1, Figure S1: Surface characterization of the electrode at different modification stages: bare ZnO electrode, after DTSSP functionalization, and after antibody immobilization; Figure S2: Calibrated dose–response plots for *Aflatoxin B1* (A) and *Salmonella Typhi* (B) in potable water, showing change in impedance response across the tested concentration range; Figure S3: Cross-reactivity analysis for AFM_1_ and AFB_1_ on AFB_1_—modified sensors tested with mixed analyte cocktails, showing minimal non-specific response. Table S1: Maximum limit of *Aflatoxin B1* by food product as per EU standards; Table S2: Comparison of the developed immunosensor with other studied label-free detection of *S. Typhi* and AFB1 [50,51,52,53,54,55,56,57,58,59,60,61,62,63,64,65,66,67,68].

## References

[1] Galán J.E. (2021). *Salmonella* Typhimurium and inflammation: A pathogen-centric affair. Nat. Rev. Microbiol..

[2] Sanderson K.E., Roth J.R. (1988). Linkage Map of *Salmonella* Typhimurium, Edition VII. Microbiol. Rev..

[3] Rushing B.R., Selim M.I. (2019). Aflatoxin B1: A review on metabolism, toxicity, occurrence in food, occupational exposure, and detoxification methods. Food Chem. Toxicol..

[4] Guengerich F., Johnson W.W., Shimada T., Ueng Y.-F., Yamazaki H., Langouët S. (1998). Activation and detoxication of aflatoxin B1. Mutat. Res. Mol. Mech. Mutagen..

[5] Rawal S., Kim J.E., Coulombe R. (2010). Aflatoxin B1 in poultry: Toxicology, metabolism and prevention. Res. Vet. Sci..

[6] Ciobanu D., Hosu-Stancioiu O., Melinte G., Ognean F., Simon I., Cristea C. (2024). Recent Progress of Electrochemical Aptasensors toward AFB1 Detection (2018–2023). Biosensors.

[7] Liao C.-M., Chen S.-C. (2005). A probabilistic modeling approach to assess human inhalation exposure risks to airborne aflatoxin B1 (AFB1). Atmos. Environ..

[8] Andrews J.R., Yu A.T., Saha S., Shakya J., Aiemjoy K., Horng L., Qamar F., Garrett D., Baker S., Saha S. (2020). Environmental Surveillance as a Tool for Identifying High-risk Settings for Typhoid Transmission. Clin. Infect. Dis..

[9] Kingsley R.A., Bäumler A.J. (2000). Host adaptation and the emergence of infectious disease: The *Salmonella* paradigm. Mol. Microbiol..

[10] Coburn B., A Grassl G., Finlay B.B. (2006). *Salmonella*, the host and disease: A brief review. Immunol. Cell Biol..

[11] Crump J.A., Heyderman R.S. (2015). A Perspective on Invasive *Salmonella* Disease in Africa. Clin. Infect. Dis..

[12] Tsougeni K., Papadakis G., Gianneli M., Grammoustianou A., Constantoudis V., Dupuy B., Petrou P.S., Kakabakos S.E., Tserepi A., Gizeli E. (2015). Plasma nanotextured polymeric lab-on-a-chip for highly efficient bacteria capture and lysis. Lab Chip.

[13] Awang M.S., Bustami Y., Hamzah H.H., Zambry N.S., Najib M.A., Khalid M.F., Aziah I., Manaf A.A. (2021). Advancement in *Salmonella* Detection Methods: From Conventional to Electrochemical-Based Sensing Detection. Biosensors.

[14] Kuhn K.G., Falkenhorst G., Ceper T.H., Dalby T., Ethelberg S., Mølbak K., Krogfelt K.A. (2012). Detecting Non-Typhoid Sal-monella in Humans by ELISAs: A Literature Review. J. Med. Microbiol..

[15] Nowak B., von Müffling T., Chaunchom S., Hartung J. (2007). *Salmonella* contamination in pigs at slaughter and on the farm: A field study using an antibody ELISA test and a PCR technique. Int. J. Food Microbiol..

[16] Cioffi A., Mancini M., Gioia V., Cinti S. (2021). Office Paper-Based Electrochemical Strips for Organophosphorus Pesticide Monitoring in Agricultural Soil. Environ. Sci. Technol..

[17] Li X., Gao X., Gai P., Liu X., Li F. (2020). Degradable metal-organic framework/methylene blue composites-based homogeneous electrochemical strategy for pesticide assay. Sens. Actuators B Chem..

[18] Liu X., Cheng H., Zhao Y., Wang Y., Li F. (2022). Portable electrochemical biosensor based on laser-induced graphene and MnO2 switch-bridged DNA signal amplification for sensitive detection of pesticide. Biosens. Bioelectron..

[19] Cheng J., Yu P., Huang Y., Zhang G., Lu C., Jiang X. (2022). Application Status and Prospect of Impedance Spectroscopy in Agricultural Product Quality Detection. Agriculture.

[20] Yu L., Zhang Y., Hu C., Wu H., Yang Y., Huang C., Jia N. (2015). Highly sensitive electrochemical impedance spectroscopy immunosensor for the detection of AFB1 in olive oil. Food Chem..

[21] Grossi M., Riccò B. (2017). Electrical impedance spectroscopy (EIS) for biological analysis and food characterization: A review. J. Sens. Sens. Syst..

[22] Mishra K.K., Dhamu V.N., Kokala A., Muthukumar S., Prasad S. (2025). Advancing food Safety: Two-plex electrochemical biosensor for mycotoxin detection in food matrices. Biosens. Bioelectron. X.

[23] Wang L., Huo X., Qi W., Xia Z., Li Y., Lin J. (2020). Rapid and sensitive detection of *Salmonella* Typhimurium using nickel nanowire bridge for electrochemical impedance amplification. Talanta.

[24] Nandakumar V., La Belle J.T., Reed J., Shah M., Cochran D., Joshi L., Alford T. (2008). A methodology for rapid detection of *Salmonella* typhimurium using label-free electrochemical impedance spectroscopy. Biosens. Bioelectron..

[25] Mishra K.K., Dhamu V.N., Jophy C., Muthukumar S., Prasad S. (2024). Electroanalytical Platform for Rapid *E. coli* O157:H7 Detection in Water Samples. Biosensors.

[26] Mishra K.K., Dhamu V.N., Poudyal D.C., Muthukumar S., Prasad S. (2024). PathoSense: A rapid electroanalytical device platform for screening *Salmonella* in water samples. Microchim. Acta.

[27] Yang L., Ruan C., Li Y. (2003). Detection of viable *Salmonella* typhimurium by impedance measurement of electrode capacitance and medium resistance. Biosens. Bioelectron..

[28] Lopez-Tellez J., Sanchez-Ortega I., Hornung-Leoni C.T., Santos E.M., Miranda J.M., Rodriguez J.A. (2020). Impedimetric Biosensor Based on a *Hechtia argentea* Lectin for the Detection of *Salmonella* spp.. Chemosensors.

[29] Zambry N.S., Awang M.S., Hamzah H.H., Mohamad A.N., Khalid M.F., Khim B.K., Bustami Y., Jamaluddin N.F., Ibrahim F., Aziah I. (2024). A portable label-free electrochemical DNA biosensor for rapid detection of *Salmonella* Typhi. Anal. Methods.

[30] Malvano F., Pilloton R., Albanese D. (2020). A novel impedimetric biosensor based on the antimicrobial activity of the peptide nisin for the detection of *Salmonella* spp.. Food Chem..

[31] Lu L., Chee G., Yamada K., Jun S. (2013). Electrochemical impedance spectroscopic technique with a functionalized microwire sensor for rapid detection of foodbornepathogens. Biosens. Bioelectron..

[32] Owino J.H.O., Ignaszak A., Al-Ahmed A., Baker P.G.L., Alemu H., Ngila J.C., Iwuoha E.I. (2007). Modelling of the impedimetric responses of an aflatoxin B1 immunosensor prepared on an electrosynthetic polyaniline platform. Anal. Bioanal. Chem..

[33] Chen L., Jiang J., Shen G., Yu R. (2014). A label-free electrochemical impedance immunosensor for the sensitive detection of aflatoxin B_1_. Anal. Methods.

[34] Lin T., Shen Y. (2020). Fabricating electrochemical aptasensors for detecting aflatoxin B1 via layer-by-layer self-assembly. J. Electroanal. Chem..

[35] Gevaerd A., Banks C.E., Bergamini M.F., Marcolino-Junior L.H. (2020). Nanomodified Screen-Printed Electrode for direct determination of Aflatoxin B1 in malted barley samples. Sens. Actuators B Chem..

[36] Spiro J.C.K., Mishra K.K., Dhamu V.N., Bhatia A., Muthukumar S., Prasad S. (2024). Development of a porElectrochemi electrochemical sensing platform for impedance spectroscopy-based biosensing using an ARM-based microcontroller. Sens. Diagn..

[37] Tanak A.S., Jagannath B., Tamrakar Y., Muthukumar S., Prasad S. (2019). Non-faradaic electrochemical impedimetric profiling of procalcitonin and C-reactive protein as a dual marker biosensor for early sepsis detection. Anal. Chim. Acta: X.

[38] Daniels J.S., Pourmand N. (2007). Label-Free Impedance Biosensors: Opportunities and Challenges. Electroanalysis.

[39] Kaminiaris M.D., Mavrikou S., Georgiadou M., Paivana G., Tsitsigiannis D.I., Kintzios S. (2020). An Impedance Based Electrochemical Immunosensor For Aflatoxin B_1_ Monitoring in Pistachio Matrices. Chemosensors.

[40] Angelopoulou M., Petrou P., Misiakos K., Raptis I., Kakabakos S. (2022). Simultaneous Detection of *Salmonella typhimurium* and *Escherichia coli O157:H7* in Drinking Water and Milk with Mach–Zehnder Interferometers Monolithically Integrated on Silicon Chips. Biosensors.

[41] Curulli A. (2021). Electrochemical Biosensors in Food Safety: Challenges and Perspectives. Molecules.

[42] Freitas M., Neves M.M.P.S., Nouws H.P.A., Delerue-Matos C. (2021). Electrochemical Immunosensor for the Simultaneous Determination of Two Main Peanut Allergenic Proteins (Ara h 1 and Ara h 6) in Food Matrices. Foods.

[43] Jiang M., Braiek M., Florea A., Chrouda A., Farre C., Bonhomme A., Bessueille F., Vocanson F., Zhang A., Jaffrezic-Renault N. (2015). Aflatoxin B1 Detection Using a Highly-Sensitive Molecularly-Imprinted Electrochemical Sensor Based on an Electropolymerized Metal Organic Framework. Toxins.

[44] CLSI Evaluation of Precision of Quantitative Measurement Procedures; Approved Guideline. CLSI Document EP05-A3; Clinical and Laboratory Standards Institute Wayne (PA). https://webstore.ansi.org/preview-pages/CLSI/preview_CLSI+EP05-A3.pdf?srsltid=AfmBOormwSYKgjARaNMMeixA3GWxnTnjfL3VgFIl7W9UKVf4yls_LBcP.

[45] Mishra K.K., Dhamu V.N., Muthukumar S., Prasad S. (2025). Quick and Sensitive Two-Plex Electrochemical Platform for Pathogen Detection in Water. Nano Sel..

[46] Florkowski C.M. (2008). Sensitivity, specificity, receiver-operating characteristic (ROC) curves and likelihood ratios: Communicating the performance of diagnostic tests. Clin. Biochem. Rev..

[47] Tieu M.-V., Choi S.H., Le H.T.N., Cho S. (2023). Electrochemical impedance-based biosensor for label-free determination of plasma P-tau181 levels for clinically accurate diagnosis of mild cognitive impairment and Alzheimer’s disease. Anal. Chim. Acta.

[48] Fawcett T. (2006). An Introduction to ROC analysis. Pattern Recogn. Lett..

[49] Mishra K.K., Thakkar K.M., Dhamu V.N., Muthukumar S., Prasad S. (2025). Electrochemical Sensor Platform for Rapid Detection of Foodborne Toxins. Biosensors.

[50] Dohlman E. (2003). Mycotoxin Hazards and Regulations.

[51] Paniel N., Radoi A., Marty J.-L. (2010). Development of an Electrochemical Biosensor for the Detection of Aflatoxin M1 in Milk. Sensors.

[52] Shim W.-B., Kim M.J., Mun H., Kim M.-G. (2014). An Aptamer-Based Dipstick Assay for the Rapid and Simple Detection of Aflatoxin B1. Biosens. Bioelectron..

[53] Sergeyeva T., Yarynka D., Piletska E., Linnik R., Zaporozhets O., Brovko O., Piletsky S., El’skaya A. (2019). Development of a Smartphone-Based Biomimetic Sensor for Aflatoxin B1 Detection Using Molecularly Imprinted Polymer Membranes. Talanta.

[54] Sun L., Wu L., Zhao Q. (2017). Aptamer Based Surface Plasmon Resonance Sensor for Aflatoxin B1. Microchim. Acta.

[55] Zhang X., Li C.-R., Wang W.-C., Xue J., Huang Y.-L., Yang X.-X., Tan B., Zhou X.-P., Shao C., Ding S.-J. (2016). A Novel Electrochemical Immunosensor for Highly Sensitive Detection of Aflatoxin B1 in Corn Using Single-Walled Carbon Nanotubes/Chitosan. Food Chem..

[56] Tan H., Ma L., Guo T., Zhou H., Chen L., Zhang Y., Dai H., Yu Y. (2019). A Novel Fluorescence Aptasensor Based on Mesoporous Silica Nanoparticles for Selective and Sensitive Detection of Aflatoxin B1. Anal. Chim. Acta.

[57] Xu M., Wang R., Li Y. (2016). Rapid Detection of *Escherichia coli* O157:H7 and *Salmonella* Typhimurium in Foods Using an Electrochemical Immunosensor Based on Screen-Printed Interdigitated Microelectrode and Immunomagnetic Separation. Talanta.

[58] Bhandari D., Chen F.C., Bridgman R.C. (2019). Detection of *Salmonella* Typhimurium in Romaine Lettuce Using a Surface Plasmon Resonance Biosensor. Biosensors.

[59] Bokken G.C.A.M., Corbee R.J., Van Knapen F., Bergwerff A.A. (2003). Immunochemical Detection of *Salmonella* Group B, D and E Using an Optical Surface Plasmon Resonance Biosensor. FEMS Microbiol. Lett..

[60] Nguyen H.H., Yi S.Y., Woubit A., Kim M. (2016). A Portable Surface Plasmon Resonance Biosensor for Rapid Detection of *Salmonella* Typhimurium. Appl. Sci. Converg. Technol..

[61] Xu Y., Luo Z., Chen J., Huang Z., Wang X., An H., Duan Y. (2018). ω-Shaped Fiber-Optic Probe-Based Localized Surface Plasmon Resonance Biosensor for Real-Time Detection of *Salmonella* Typhimurium. Anal. Chem..

[62] Seo K.H., Brackett R.E., Hartman N.F., Campbell D.P. (1999). Development of a Rapid Response Biosensor for Detection of *Salmonella* Typhimurium. J. Food Prot..

[63] Das R.D., RoyChaudhuri C., Maji S., Das S., Saha H. (2009). Macroporous Silicon Based Simple and Efficient Trapping Platform for Electrical Detection of *Salmonella* Typhimurium Pathogens. Biosens. Bioelectron..

[64] Farka Z., Juřík T., Pastucha M., Kovář D., Lacina K., Skládal P. (2016). Rapid Immunosensing of *Salmonella* Typhimurium Using Electrochemical Impedance Spectroscopy: The Effect of Sample Treatment. Electroanalysis.

[65] Kaushik S., Pandey A., Tiwari U.K., Sinha R.K. (2018). A Label-Free Fiber Optic Biosensor for *Salmonella* Typhimurium Detection. Opt. Fiber Technol..

[66] Wang H., Zhao Y., Bie S., Suo T., Jia G., Liu B., Ye R., Li Z. (2019). Development of an Electrochemical Biosensor for Rapid and Effective Detection of Pathogenic *Escherichia coli* in Licorice Extract. Appl. Sci..

[67] Housaindokht M.R., Sheikhzadeh E., Pordeli P., Rouhbakhsh Zaeri Z., Janati-Fard F., Nosrati M. (2018). Mashreghi, M.; Nakhaeipour, A.; A. Esmaeili, A.; Solimani, S. A Sensitive Electrochemical Aptasensor Based on Single Wall Carbon Nanotube Modified Screen Printed Electrode for Detection of *Escherichia coli* O157:H7. Adv. Mater. Lett..

[68] Guo Y., Wang Y., Liu S., Yu J., Wang H., Cui M., Huang J. (2015). Electrochemical Immunosensor Assay (EIA) for Sensitive Detection of *E. coli* O157:H7 with Signal Amplification on a SG-PEDOT-AuNPs Electrode Interface. Analyst.

