# Supplementary material for: Duplex EIS Sensor for Salmonella Typhi and Aflatoxin B1 Detection in Soil Runoff
Source: Biosensors (Basel). 2025 Oct 1;15(10):654. doi: 10.3390/bios15100654 (PMC12562585; doi:10.3390/bios15100654)
Supplement: Supplementary file 1 [file biosensors-15-00654-s001.zip › biosensors-3849982-supplementary.pdf]

biosensors

MDPI

Supplementary Materials

Duplex EIS Sensor for *Salmonella Typhi* and *Aflatoxin B1* Detection in Soil Runoff

Kundan Kumar Mishra [1], Krupa M Thakkar [1], Sumana Karmakar [1], Vikram Narayanan Dhamu [2], Sriram Muthukumar [2] and Shalini Prasad [1,2,*]

[1] Department of Bioengineering, University of Texas at Dallas, Richardson, TX 75080, USA,
[2] EnLiSense LLC, 1813 Audubon Pondway, Allen, TX 75013, USA.
* Correspondence: shalini.prasad@utdallas.edu

Table S1. Maximum limit of *Aflatoxin B1* by food product as per EU standards [1].

**Food Product**	**Maximum Limit (ppb)**
Peanuts, dried fruit, nuts for human consumption	2
Peanuts before being processed or peanuts added as an ingredient to a food for human consumption	8
Nuts or dried fruit before being processed or peanuts added as an ingredient to a food for human consumption	5
Cereals and their processed form or as an ingredient in food for human consumption	2
Feed material from peanuts, palm-kernel, corn, copra, cottonseed, and their processed forms	20
Feed for dairy cattle	5
Feed for mature pigs and poultry	20

Table S2. Comparison of the developed immunosensor with other studied label-free detection of *S. Typhi* and AFB1.

**Device Type/ Detection Method**	**Mycotoxin Detected**	**Sample Type**	**LoD**	**Assay Time**	**Reference**
Chronoamperometry	*Aflatoxin*	Milk	0.01 ppb	15 minutes for sample preparation<br>Time for reading not given	[2]
Aptameric	*Aflatoxin*	Corn	0.1 ng/mL	20 minutes for sample preparation<br>20 minutes for incubation and reading time	[3]
Fluorescence (Smartphone Camera)	*Aflatoxin*	Maize	20 ng/mL	16 minutes sample preparation<br>1 minute for reading	[4]

Aptameric	*Aflatoxin*	Red wine and Beer	0.4 nM	13 minutes sensor preparation time<br>6 minutes total runtime for reading	[5]
Cyclic Voltammetry and Square Wave Voltammetry	*Aflatoxin*	Corn Powder	3.5 pg/mL	15.5 hours for sensor and sample preparation<br>1.5+ hours total runtime for reading	[6]
Aptameric with Fluorescence	*Aflatoxin*	Corn + peanut + oatmeal + rice mixture<br>And<br>Peanut Oil + Corn Oil mixture	0.13 ng/mL	41.5+ hours for sensor and sample preparation<br>Time for reading not given	[7]
EIS	*S. Typhi*	chicken rinse water	3.4×10^2	105	[8]
SPR	*S. Typhi*	romaine lettuce	4.7×10^5	<6	[9]
SPR	*S. Typhi*	buffer	1.7×10^3	22	[10]
Portable SPR	*S. Typhi*	buffer	10^7	60	[11]
Ω-shaped fiber-optic LSPR	*S. Typhi*	buffer	<128	100	[12]
Hartman interferometry	*S. Typhi*	assay buffer	10^4	10	[13]
EIS	*S. Typhi*	buffer	1×10^3	30	[14]
EIS	*S. Typhi*	Milk	1×10^3	20	[15]
Fiber-optic	*S. Typhi*	Milk	247	100	[9]
DPV	*S. Typhi*	Licorice Extract	80	150	[17]
CV–DPV	*S. Typhi*	Water	17	35	[18]
DPV	*S. Typhi*	spring water and milk	34	150	[19]
EIS	*S. Typhi* and *ABF1*	Soil run-off	1CFU/mL,<br>0.001ng/mL	5	This work

SPR-Surface plasmon resonance; EIS-Electrochemical impedance spectroscopy.

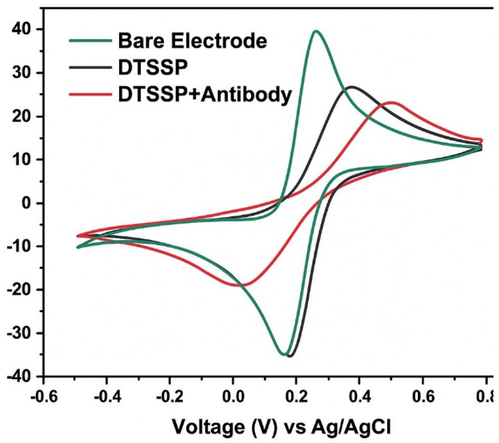

Figure S1. Surface characterization of the electrode at different modification stages: bare ZnO electrode, after DTSSP functionalization, and after antibody immobilization. The changes in surface morphology and electrochemical behavior confirm successful stepwise modification.

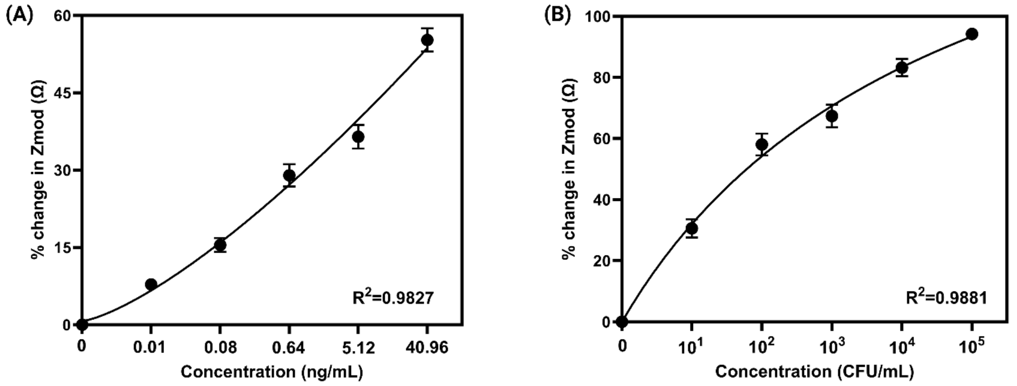

Figure S2. Calibrated dose–response plots for *Aflatoxin B1* (A) and *Salmonella Typhi* (B) in potable water, showing change in impedance response across the tested concentration range.

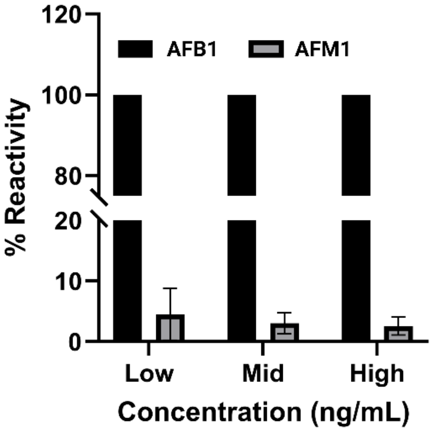

Figure S3. Cross-reactivity analysis for AFM_1 and AFB_1 on AFB_1 - modified sensors tested with mixed analyte cocktails, showing minimal non-specific response.

References

1. Dohlman, E. *Mycotoxin Hazards and Regulations*; U.S Department of Agriculture, 2003;
2. Paniel, N.; Radoi, A.; Marty, J.-L. Development of an Electrochemical Biosensor for the Detection of Aflatoxin M1 in Milk. *Sensors* **2010**, *10*, 9439–9448, doi:10.3390/s101009439.
3. Shim, W.-B.; Kim, M.J.; Mun, H.; Kim, M.-G. An Aptamer-Based Dipstick Assay for the Rapid and Simple Detection of Aflatoxin B1. *Biosens Bioelectron* **2014**, *62*, 288–294, doi:10.1016/j.bios.2014.06.059.
4. Sergeyeva, T.; Yarynka, D.; Piletska, E.; Linnik, R.; Zaporozhets, O.; Brovko, O.; Piletsky, S.; El'skaya, A. Development of a Smartphone-Based Biomimetic Sensor for Aflatoxin B1 Detection Using Molecularly Imprinted Polymer Membranes. *Talanta* **2019**, *201*, 204–210, doi:10.1016/j.talanta.2019.04.016.
5. Sun, L.; Wu, L.; Zhao, Q. Aptamer Based Surface Plasmon Resonance Sensor for Aflatoxin B1. *Microchimica Acta* **2017**, *184*, 2605–2610, doi:10.1007/s00604-017-2265-5.
6. Zhang, X.; Li, C.-R.; Wang, W.-C.; Xue, J.; Huang, Y.-L.; Yang, X.-X.; Tan, B.; Zhou, X.-P.; Shao, C.; Ding, S.-J.; et al. A Novel Electrochemical Immunosensor for Highly Sensitive Detection of Aflatoxin B1 in Corn Using Single-Walled Carbon Nanotubes/Chitosan. *Food Chem* **2016**, *192*, 197–202, doi:10.1016/j.foodchem.2015.06.044.
7. Tan, H.; Ma, L.; Guo, T.; Zhou, H.; Chen, L.; Zhang, Y.; Dai, H.; Yu, Y. A Novel Fluorescence Aptasensor Based on Mesoporous Silica Nanoparticles for Selective and Sensitive Detection of Aflatoxin B1. *Anal Chim Acta* **2019**, *1068*, 87–95, doi:10.1016/j.aca.2019.04.014.
8. Xu, M.; Wang, R.; Li, Y. Rapid Detection of Escherichia Coli O157:H7 and Salmonella Typhimurium in Foods Using an Electrochemical Immunosensor Based on Screen-Printed Interdigitated Microelectrode and Immunomagnetic Separation. *Talanta* **2016**, *148*, 200–208, doi:10.1016/j.talanta.2015.10.082.
9. Bhandari, D.; Chen, F.C.; Bridgman, R.C. Detection of Salmonella Typhimurium in Romaine Lettuce Using a Surface Plasmon Resonance Biosensor. *Biosensors (Basel)* **2019**, *9*, doi:10.3390/bios9030094.
10. Bokken, G.C.A.M.; Corbee, R.J.; Van Knapen, F.; Bergwerff, A.A. Immunochemical Detection of Salmonella Group B, D and E Using an Optical Surface Plasmon Resonance Biosensor. *FEMS Microbiol Lett* **2003**, *222*, 75–82, doi:10.1016/S0378-1097(03)00250-7.
11. Nguyen, H.H.; Yi, S.Y.; Woubit, A.; Kim, M. A Portable Surface Plasmon Resonance Biosensor for Rapid Detection of Salmonella Typhimurium. *Applied Science and Convergence Technology* **2016**, *25*, 61–65, doi:10.5757/asct.2016.25.3.61.
12. Xu, Y.; Luo, Z.; Chen, J.; Huang, Z.; Wang, X.; An, H.; Duan, Y. ω-Shaped Fiber-Optic Probe-Based Localized Surface Plasmon Resonance Biosensor for Real-Time Detection of Salmonella Typhimurium. *Anal Chem* **2018**, *90*, 13640–13646, doi:10.1021/acs.analchem.8b03905.
13. Seo, K.H.; Brackett, R.E.; Hartman, N.F.; Campbell, D.P. Development of a Rapid Response Biosensor for Detection of Salmonella Typhimurium. *J Food Prot* **1999**, *62*, 431–437, doi:10.4315/0362-028X-62.5.431.
14. Das, R.D.; RoyChaudhuri, C.; Maji, S.; Das, S.; Saha, H. Macroporous Silicon Based Simple and Efficient Trapping Platform for Electrical Detection of Salmonella Typhimurium Pathogens. *Biosens Bioelectron* **2009**, *24*, 3215–3222, doi:10.1016/j.bios.2009.04.014.
15. Farka, Z.; Juřík, T.; Pastucha, M.; Kovář, D.; Lacina, K.; Skládal, P. Rapid Immunosensing of Salmonella Typhimurium Using Electrochemical Impedance Spectroscopy: The Effect of Sample Treatment. *Electroanalysis* **2016**, *28*, 1803–1809, doi:10.1002/elan.201600093.
16. Kaushik, S.; Pandey, A.; Tiwari, U.K.; Sinha, R.K. A Label-Free Fiber Optic Biosensor for Salmonella Typhimurium Detection. *Optical Fiber Technology* **2018**, *46*, 95–103, doi:https://doi.org/10.1016/j.yofte.2018.09.012.
17. Wang, H.; Zhao, Y.; Bie, S.; Suo, T.; Jia, G.; Liu, B.; Ye, R.; Li, Z. Development of an Electrochemical Biosensor for Rapid and Effective Detection of Pathogenic Escherichia Coli in Licorice Extract. *Applied Sciences (Switzerland)* **2019**, *9*, doi:10.3390/app9020295.
18. Housaindokht, M.R.; Sheikhzadeh, E.; Pordeli, P.; Rouhbakhsh Zaeri, Z.; Janati-Fard, F.; Nosrati M. Mashreghi, M.; Nakhaeipour, A.; A. Esmaeili, A.; Solimani, S. A Sensitive Electrochemical Aptasensor Based on Single Wall Carbon Nanotube Modified Screen Printed Electrode for Detection of Escherichia Coli O157:H7. *Adv Mater Lett* **2018**, *9*, 369–374, doi:10.5185/amlett.2018.1701.
19. Guo, Y.; Wang, Y.; Liu, S.; Yu, J.; Wang, H.; Cui, M.; Huang, J. Electrochemical Immunosensor Assay (EIA) for Sensitive Detection of E. Coli O157:H7 with Signal Amplification on a SG-PEDOT-AuNPs Electrode Interface. *Analyst* **2015**, *140*, 551–559, doi:10.1039/c4an01463d.
